# From biochemical analysis to targeted therapies

**DOI:** 10.1186/1755-1536-5-S1-S1

**Published:** 2012-06-06

**Authors:** Petro E Petrides, David Brenner

**Affiliations:** 1 Hematology Oncology Center & Ludwig Maximilians University Medical School, Munich, Germany; 2 University of California, San Diego, La Jolla, CA, USA

## Introduction

Abnormal fibrogenesis is a process which in practically every organ of our body can contribute to disease. In contrast to its paramount importance, too little is known about the pathophysiology of this process and therapeutical ways to interfere with this process.

In order to spark progress in the field our idea was to bring together a small group of leading experts of tissue fibrogenesis to focus on this topic by investigating different tissues and diseases. Cross fertilization through intense discussion should allow us to work out general principles and develop new strategies for targeted therapies.

For the venue we chose the beautiful island of Frauenchiemsee in Upper Bavaria where international meetings on various topics had been held over the last decade. Leading experts had met to discuss the role of peroxidases (1998), chronic myeloproliferative disorders (2000), iron metabolism (2003) and thrombus formation (2005). For details the interested reader is referred to www.innova-med.de.

**Figure 1:**
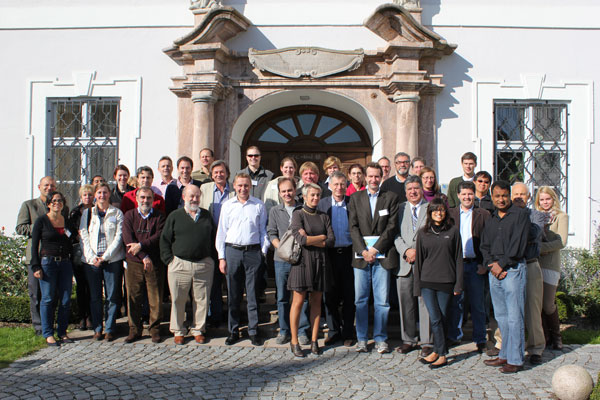
**Group photo**.

On this secluded island in beautiful surroundings a group of 30 experts (see Figure 1) in the field came together in fall 2010 for a few days to catalyze interpersonal communication, to stimulate the fruitful exchange of ideas and foster future collaborations.

The meeting started with basic concepts, continued with fibrosis in various organs, and went on to the reversibility of this process and the development of antifibrotic therapies. Although each organ is unique, all fibrotic diseases share core pathogenic pathways. For this publication in *Fibrogenesis & Tissue Repair *all authors have updated their presentations.

The meeting was financially made possible as in the past through the support of a peer reviewed grant from the Deutsche Forschungsgemeinschaft (DFG), Bonn, Germany and additional funds from AOP Orphan Pharmaceuticals, Vienna, Austria as well as private contributions.

We hope that the reader will enjoy these proceedings and gain some inspiration for the research in his/her own area of interest.

## Competing interests

The authors declare that they have no competing interests.

